# Inherited Defects of the ASC-1 Complex in Congenital Neuromuscular Diseases

**DOI:** 10.3390/ijms22116039

**Published:** 2021-06-03

**Authors:** Justine Meunier, Rocio-Nur Villar-Quiles, Isabelle Duband-Goulet, Ana Ferreiro

**Affiliations:** 1 Basic and Translational Myology Laboratory, UMR8251, University of Paris/National Center for Scientific Research, 75013 Paris, France; justine.meunier@etu.u-paris.fr (J.M.); rocionur.villarquiles@aphp.fr (R.-N.V.-Q.); 2 Reference Center for Neuromuscular Disorders, Pitié-Salpêtrière Hospital, APHP, Institute of Myology, 75013 Paris, France

**Keywords:** congenital myopathies, spinal muscular atrophy, *TRIP4*, *ASCC1*, cell cycle, cell proliferation, muscle growth, arthrogryposis multiplex congenita, antenatal bone fractures, ASC-1 complex

## Abstract

Defects in transcriptional and cell cycle regulation have emerged as novel pathophysiological mechanisms in congenital neuromuscular disease with the recent identification of mutations in the *TRIP4* and *ASCC1* genes, encoding, respectively, ASC-1 and ASCC1, two subunits of the ASC-1 (Activating Signal Cointegrator-1) complex. This complex is a poorly known transcriptional coregulator involved in transcriptional, post-transcriptional or translational activities. Inherited defects in components of the ASC-1 complex have been associated with several autosomal recessive phenotypes, including severe and mild forms of striated muscle disease (congenital myopathy with or without myocardial involvement), but also cases diagnosed of motor neuron disease (spinal muscular atrophy). Additionally, antenatal bone fractures were present in the reported patients with *ASCC1* mutations. Functional studies revealed that the ASC-1 subunit is a novel regulator of cell cycle, proliferation and growth in muscle and non-muscular cells. In this review, we summarize and discuss the available data on the clinical and histopathological phenotypes associated with inherited defects of the ASC-1 complex proteins, the known genotype–phenotype correlations, the ASC-1 pathophysiological role, the puzzling question of motoneuron versus primary muscle involvement and potential future research avenues, illustrating the study of rare monogenic disorders as an interesting model paradigm to understand major physiological processes.

## 1. Introduction

Congenital myopathies (CMs) are a group of rare inherited muscle disorders, genetically and clinically heterogeneous. They usually present at birth or in early childhood with delayed motor development and static or slowly progressive muscular weakness and hypotonia [1]. CMs are often associated with cardiac and/or respiratory failure [2,3,4] and most of them have no specific treatment so far [5]. The hallmark of congenital myopathies is the presence of characteristic abnormalities in the internal architecture of muscle fibers, typically without significant dystrophic lesions (necrosis, regeneration or fibrosis); dystrophic changes are the hallmark of congenital muscular dystrophies, a different nosological group. The type of structural lesion observed in muscle biopsies defines each individual congenital myopathy. For instance, the presence of sarcoplasmic and/or nuclear rod-shaped inclusions is characteristic of nemaline myopathy; focal areas of sarcomere disorganization and mitochondria depletion (cores) identify core myopathies; abundant internalized (rather than peripheral/subsarcolemmal) nuclei are observed in centronuclear myopathies; and subsarcolemmal croissant-shaped areas of myofilament disarray (termed caps) define cap disease.

Next-generation sequencing has allowed the identification of a growing number of genes associated with CMs. Most of these genes encode proteins involved in the architecture or the contractile function of muscle fibers, including calcium homeostasis [6], excitation-contraction coupling [7], membrane remodeling [8,9], myofibrillar force generation [10,11] or thin-thick filament assembly [12] and interactions [13]. More than 30 genes have been associated with CMs; most individual CMs show a remarkable genetic heterogeneity and phenotypical overlap with other subtypes, blurring the classically established boundaries [3,14,15]. However, a significant proportion of CM patients (up to 40% cases [16,17]) remain genetically uncharacterized.

Defects in transcriptional or post-transcriptional regulation had not been associated with human neuromuscular diseases until recent times. We reported five years ago a novel and severe recessive congenital myopathy with core lesions in a large consanguineous family presenting with a so-far unreported histological phenotype [18]. The causative gene identified, *TRIP4* (Thyroid Hormone Receptor Interactor 4), encodes a poorly known transcriptional co-activator, ASC-1 (Activating Signal Cointegrator-1). ASC-1 is predicted to contain two functional domains: a zinc finger motif interacting in vitro with basal transcription factors, transcription integrators or nuclear receptors [19] and a ASCH domain potentially binding RNA [20,21]. Lately, we found that ASC-1 is a novel regulator of cell cycle, cell proliferation and muscle cell growth [21], key mechanisms in myogenesis. Thus, transcriptional and cell cycle regulation have emerged as novel mechanisms and pathophysiological pathways in congenital muscle disease, and ASC-1 as a pivotal actor in skeletal muscle physiology [18,22]. Since ASC-1 interacts with three protein partners in a complex called the ASC-1 complex [19], genes encoding other proteins of this complex represented potential candidate genes for congenital muscle disease. This was confirmed by the identification of mutations in the gene *ASCC1*, encoding the ASC-1 partner protein ASCC1 (Activating Signal Cointegrator 1 Complex Subunit 1) [23].

Unexpectedly, defects in components of the ASC-1 complex seem to lead to an unusually large spectrum of neuromuscular diseases. Mutations of *TRIP4* or *ASCC1* have been reported both in patients with a congenital myopathy without any sign of motor neuron involvement, and in patients with a diagnosis of congenital motor neuron disease (Spinal Muscular Atrophy, SMA) associated with prenatal bone fractures [23,24]. Indeed, the ASC-1 complex was recently proposed to play a relevant role in congenital and degenerative motor neuron diseases and to represent a common link between SMA and the neurodegenerative disease amyotrophic lateral sclerosis (ALS) [25]. Thus, the ASC-1 complex is attracting increasing attention beyond the study of rare diseases, both on the neuromuscular and the basic science fields.

The pathomechanisms underlying congenital and degenerative motor neuron and muscle diseases being far from fully understood, which hinders their potential use as therapeutic targets, the study of ASC-1 related diseases might represent a useful model paradigm. We will review here the available data on their clinical and histopathological phenotype, genotype–phenotype correlations, the role of ASC-1 as a new regulator of cell cycle and muscle cell growth and the puzzling question of motor neuron versus primary muscle involvement.

## 2. *TRIP4* Mutations Are Associated with a Large Spectrum of Clinical and Histological Phenotypes, Potentially Affecting Cardiac Muscles

In 2016, we studied four patients from a large consanguineous family who presented with severe neonatal hypotonia and muscle weakness. The latter affected limb muscles, leading to failure to acquire independent ambulation, as well as trunk and neck muscles, leading to severe scoliosis and to respiratory failure causing death in infancy in the absence of assisted ventilation. Patients showed no or little joint contractures but joint hyperlaxity and skin abnormalities (hyperkeratosis, hyperelasticity). Using a combination of positional cloning and high-throughput exome studies, we found that the affected family members were homozygous for a recessive nonsense mutation (c.950G>A, p.Trp297Ter) of the *TRIP4* gene, encoding the ASC-1 protein. Thus, we coined the term ASC-1 related myopathy (ASC1-RM) for this novel phenotypical and molecular entity [18]. A recent study of five additional families with seven novel *TRIP4* mutations [22] has expanded the phenotypical spectrum of this disease beyond very severe congenital forms, to include mild ambulatory adult patients. Overall, the clinical phenotype is marked by early-onset weakness of axial (neck and trunk) and proximal limb muscles, progressive scoliosis—sometimes associated with rigid spine—dysmorphic features and skin abnormalities reminiscent of those observed in collagen VI-related myopathies [26]. Variable respiratory involvement correlates with disease severity. Extraocular muscle involvement can be present in some patients. Electromyography studies performed in several patients from these six families revealed pure myogenic patterns, with no neurogenic findings. Muscle MRI of the lower limbs in *TRIP4*-mutant patients revealed predominant involvement of the posterior compartment of the thigh and relative preservation of semitendinosus, gracilis and sartorius [18,22]. Interestingly, cardiac involvement, including adult-onset cardiomyopathy, is part of the clinical spectrum of the disease [22], consistently with a primary involvement of striated muscles due to ASC-1 defects.

**Figure 1:**
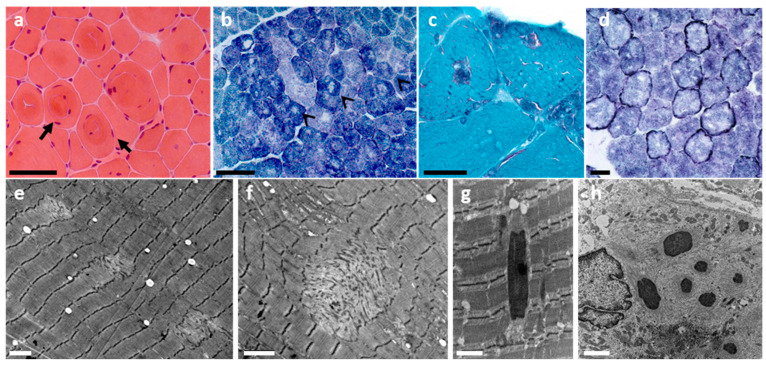
Histological spectrum of ASC1-related myopathy. Note histological features typical of a congenital myopathy such as fiber size variation (**a**,**b**), internalized nuclei (**a**) or whorled fibers (**a**, black arrows). Multi-minicores are the main histological features and can be seen as multiple lighter areas lacking oxidative activity on oxidative staining (black arrowheads in **b**) and areas of mitochondrial depletion and sarcomere disorganization on electron microscopy (EM; **e**,**f**). Modified Gomori trichrome revealed in some patients multiple inclusions (**c**), which corresponded to electron-dense nemaline rods (**g**) on EM. Intense oxidative rims beneath the sarcolemma (**d**, darker peripheral staining), compatible with mitochondrial proliferation or mislocalization, can be observed in some patients with *TRIP4* mutations and are comparable to those reported in *ASCC1*-mutated patients. Subsarcolemmal myofibrillar disorganization (sometimes forming cap lesions), cytoplasmic bodies and/rods can also be found (**h**). Transversal frozen sections: hematoxylin and eosin (**a**), NADH-TR (**b**,**d**), modified Gomori trichrome (**c**), EM (**e**–**h**). Scale bars = 50 μm (**a**,**b**), 25 μm (**c**,**d**), 2 μm (**e**,**f**,**h**), 1 μm (**g**). This figure has been modified from Ann Neurol 2020;87(2):217–232 and reproduced with permission from the publisher.

From the histopathological point of view, the spectrum of muscle fiber architectural lesions associated with *TRIP4* mutations is particularly large (Figure 1). Short core lesions termed multi-minicores are almost constantly observed, but they can coexist with cap lesions, nemaline or cytoplasmic bodies and mild dystrophic lesions [18,22]. Furthermore, rimmed fibers (with intense oxidative rims beneath the sarcolemma) have also been observed in *TRIP4*-mutant patients [21]. Thus, there is a clinical and histopathological overlap with other early-onset primary muscle disorders such as other forms of multi-minicore disease including SELENON-related myopathy [27,28,29,30], nemaline myopathy, cytoplasmic body myopathy, cap myopathy [31,32], or collagen VI-related congenital muscular dystrophy [26].

It is therefore intriguing that, simultaneously to the first report of ASC1-RM, Knierim et al. reported recessive *TRIP4* mutations associated with an antenatal phenotype diagnosed as spinal muscular atrophy (SMA) [23]. Five patients from three families presented with a phenotype marked by arthrogryposis multiplex congenita, neonatal respiratory distress requiring assisted ventilation and congenital bone fractures. Muscles did not react upon stimulation and neurography reportedly showed axonal neuropathy. Most children died from respiratory failure or acute heart failure within the first 16 months of life. Indeed, congenital heart defects (atrial septal defect, patent ductus arteriosus), a rare finding in congenital SMA, were associated in two families with cardiomyopathy; the latter is frequently associated with primary diseases of striated muscles but, to our knowledge, has not been reported in SMA patients.

Skeletal muscle biopsies from some of these children showed major reduction in and variability of myofiber size, suggesting that a primary muscle component in this second ASC-1 associated phenotype cannot be excluded. However, a tendency to clustering of type I muscle fibers (considered a sign of denervation) and the presence of apoptotic α-motoneurons in the anterior horn of the spinal cord in one patient were compatible with SMA. Sural nerve biopsies from one child showed normal density of myelinated fibers but loss of unmyelinated axons (mostly carrying autonomic or sensory information). Interestingly, *trip4*- and *ascc1*-knocked-down zebrafish models revealed severe disorganization of both myotomes and neuromuscular endplates [23].

No abnormality in brain imaging studies has been reported in patients with *TRIP4* mutations so far.

## 3. *ASCC1* Mutations: Lethal Involvement of Central Nervous System, Skeletal Muscles and Bones

Knierim et al. also reported the first mutation in the *ASCC1* gene, encoding the ASC-1 partner ASCC1, in two siblings. Their severe clinical presentation was marked by prenatal manifestations (decreased fetal movements, polyhydramnios), premature birth, severe neonatal hypotonia, areflexia, respiratory distress, difficult feeding, multiple congenital contractures (arthrogryposis multiplex congenita) and congenital bone fractures. Both patients also exhibited cardiac defects such as patent ductus arteriosus and patent foramen ovale but, although both *trip4* and *ascc1* are expressed in cardiac muscles in zebrafish, they had no myocardial disease. These patients were diagnosed as having a severe antenatal-onset SMA and died within the first months of life due to respiratory failure. Cerebral MRI performed in both siblings revealed a simplified gyral pattern of the cerebral cortex. Muscle biopsy from one of these patients showed fiber size variation, atrophic fibers and muscle fiber immaturity (intense staining for MHC_dev_). Sural biopsy from one patient showed normal density of myelinated fibers but unmyelinated axon loss [23].

Interestingly, Böhm et al. reported three additional families with *ASCC1* recessive nonsense or frameshift mutations presenting with the same lethal clinical phenotype. The muscle biopsies from these patients disclosed myopathic features (i.e., fiber size variability, myofibrillar disorganization and enlargement of the Z-bands on electron microscopy) and their EMG studies were not suggestive of motor neuron involvement but of a severe form of congenital myopathy [24]. Rimmed fibers comparable to those associated with *TRIP4* mutations were observed in patients with *ASCC1* mutations [24]. These findings suggested that *TRIP4* and *ASCC1* are implicated in a common pathophysiological pathway that leads to multiple forms of myofibrillar disarray, and thus to an overlap of different histopathological CM lesions.

The question of primary motor neuron versus muscle involvement has not been clarified by more recent reports of three other patients with 5 novel *ASCC1* mutations. Their clinical presentation was consistent with the lethal perinatal phenotype described above, including congenital bone fractures [33,34,35], mild brain ventricle dilatation abnormalities in one patient [35] and one stillbirth [24]. The muscle biopsy from one of them was reported as atrophic with no further description [33] and no muscle biopsies were performed in the remaining two cases. Dysmorphic features and a myopathic appearance were observed in most of these cases [33,35]. Although some of these patients were labeled as having SMA by analogy with the first cases with *ASCC1* mutations reported, no electrophysiological study or in-depth histopathological examination was performed supporting or clarifying this diagnosis.

## 4. Genetic Bases of the ASC-1 and ASCC1 Related Disorders

*TRIP4* is a 67.5 kbp gene localized in chromosome 15, contains 13 exons and encodes the ubiquitous 65 kDa transcriptional co-activator protein ASC-1. So far, 10 mutations in the *TRIP4* gene have been reported in 16 patients from 9 families. Most of them are frameshift mutations but one missense variant and one homozygous deletion of exons 8–9, predicting loss of ASC-1 functional domains, have also been identified [22]. Transmission was autosomal recessive in all cases, with no de novo mutations (Table 1). All heterozygous parents were asymptomatic for CNS or neuromuscular disease.

**Figure 2:**
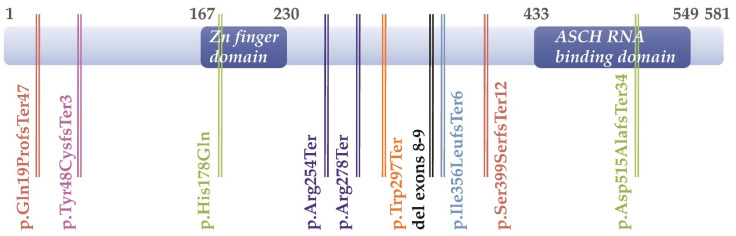
Schematic representation of the ASC-1 protein and localization of the patients’ mutations regarding the two predicted functional domains (deep blue boxes).

Mutations are distributed along the entire length of the gene (Figure 2). Some of them affect the predicted functional domains of the protein. For instance, the p.His178Gln substitution lies in the central Zinc Finger domain, while some deletions disrupt the ASCH domain located in the carboxy-terminal part (p.Asp515AlafsTer34; p.Ser399SerfsTer12; p.Ile356LeufsTer6; homozygous deletion of exons 8 + 9). Other frameshift mutations were predicted to lead to very short truncated proteins lacking any functional domain (p.Gln19ProfsTer47; p.Tyr48CysfsTer3), while three nonsense mutations were compatible with the synthesis of truncated proteins preserving at least one of the two functional domains (p.Trp297Ter; p.Arg254Ter; p.Arg278Ter). However, experimental studies using patient-derived samples disclosed ASC-1 severe depletion or absence associated with 6 of these mutations, including the *TRIP4* missense mutation (p.His178Gln), suggesting elimination by transcriptional or post-transcriptional quality-control mechanisms. Indeed, in the first ASC1-RM family reported, patients homozygous for the non-sense *TRIP4* mutation c. 950G>A (NM_016213.4) or p.Trp297Ter (NP_057297.2), which introduces a premature termination codon, showed mRNA decay and absence of protein expression. Interestingly, ASC-1 was undetectable both in the mildest and the first severe cases reported [18,22], suggesting modulators of phenotype severity other than the amount of ASC-1 protein present. Along these lines, two *TRIP4* nonsense mutations resulted in loss of the full-length protein but also, through corrective splicing events (exon skipping), in upregulation of a shorter isoform containing most ASC-1 functional domains. In spite of this, homozygous or compound heterozygous patients with these mutations (originating from the Balkan region) had a very severe neonatal neuromuscular phenotype, leading to death in the first weeks of life [23].

The *ASCC1* gene, located on chromosome 10, contains 13 exons (named 1–2, 3a, 3b, 4–8, 9a, 9b, 9c and 10) of which 11 are coding, generating multiple alternatively spliced transcripts which encode more than 20 isoforms. The transcript encoding the 41 kDa ASCC1 is the most abundant. Exon 3b is only marginally expressed according to brain and muscle RNAseq datasets [23]. Up to date, 7 mutations in the *ASCC1* gene have been identified in a total of 12 patients from 7 families. All are nonsense or frameshift variants leading to autosomal recessive phenotypes in patients. These include homozygous nonsense mutations (two mutations, four families), as well as compound heterozygosity of a nonsense mutation with either hemizygous deletions (two families) or duplication of a single nucleotide (one family). A single-nucleotide duplication in exon 3 (c.157dupG, p.Glu53GlyfsTer19) is the most frequently reported mutation. It has been identified so far in four families originating from the Mediterranean area (Turkey, Portugal, Tunisia and Morocco), both in homozygosity and in compound heterozygosity. This variant is predicted to generate a premature termination codon compatible with elimination of the mutant transcript through nonsense mediated decay. Indeed, complete absence of ASCC1 protein has been experimentally confirmed in one patient homozygous for the c.157dupG [23] (Table 2). 

Regarding potential genotype–phenotype correlations, two of the families reported by Knierim et al. as having a severe form of SMA with congenital bone fractures [23] carried *TRIP4* nonsense mutations resulting in exon skipping and upregulation of a shorter isoform containing most ASC-1 functional domains (Figure 2). By contrast, *TRIP4* mutations associated with a congenital myopathy phenotype [18,22] lead to ASC-1 protein depletion. Although it cannot be excluded that a component of primary muscle involvement contributed to the severe phenotype in the *TRIP4* mutant patients reported in [23], these genetic data suggest that absence of ASC-1 (haploinsufficiency) might be associated with a primary striated muscle phenotype, whereas expression of a shorter ASC-1 protein might lead to a partially different pathophysiological cascade, including preferential motor neuron involvement. Detailed analyses and deep phenotyping of additional patients with different *TRIP4* mutations are necessary to clarify this point. As mentioned above, absence of ASC-1 protein has been observed in both mild and severe cases and therefore does not seem to correlate with phenotype severity.

In the case of *ASCC1* mutations, no genotype–phenotype correlation can be established so-far. All mutations lead to a predicted total absence of ASCC1 protein and all patients share the severe congenital phenotype described above, lethal in the perinatal period, associated with congenital bone fractures.

## 5. Different ASC-1 Complex Conformations Reveal Specific Roles of ASC-1 and ASCC1

The ASC-1 complex was first described as a tetrameric protein complex containing four protein subunits, ASC-1, ASCC1, ASCC2 and ASCC3. Few structural and functional data are available on these proteins. As mentioned above, ASC-1, which gives its name to the complex, is predicted to contain two functional domains (Figure 2) potentially involved in protein-protein interactions or RNA binding [19,20]. The smallest partner is the ASCC1 protein (45 kDa), that contains an RNA binding K-homology (KH) domain fused to a C-terminal RNA ligase-like domain [36]. The ASCC2 (Activating Signal Cointegrator 1 Complex Subunit 2) partner is a 100 kDa subunit shown to contain a coupling of ubiquitin to ER degradation (CUE) domain [37] suspected to be involved in ubiquitin signaling. ASCC3 (Activating Signal Cointegrator 1 Complex Subunit 3) is the largest subunit (200 kDa), with a strong homology with DExH-type RNA helicase domains. However, it has recently been characterized as a DNA helicase involved in DNA damage repair [38]. The stochiometric association of the four subunits forming the ASC-1 complex in vitro was first shown to transactivate a class of transcription factors, namely AP-1, NF-kB and Serum Response Factor (SRF), involved in cell-fate controlling pathways, including proliferation, differentiation, migration or cell survival. ASC-1 was shown to be critical for the functional interaction between the ASC-1 complex and those three transcription factors through its N-terminal part, and ASCC1 was essential for AP-1 transactivation [19]. Three years ago, new transcriptional and post-transcriptional activities of this complex were discovered, revealing its contribution to general transcription and splicing events through interactions with the RNAP II machinery. Again, the ASC-1 subunit plays an essential role as this interaction was disrupted by the deletion of its C-terminal part [25].

**Figure 3:**
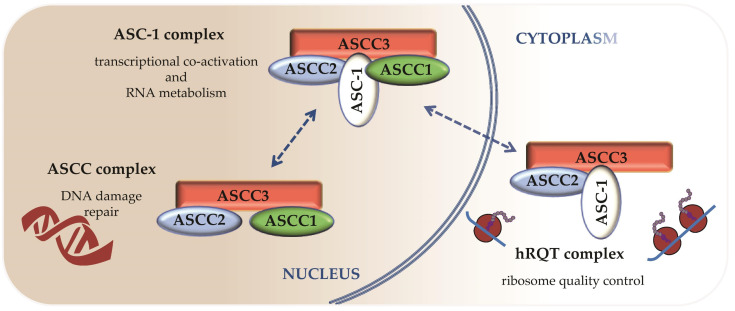
Proposed model for the different complexes containing ASC-1, ASCC1, ASCC2 and ASCC3 subunits and their distribution in cell compartments. Dashed double arrows indicate potential localization or composition changes that are yet to be demonstrated.

Interestingly, the subunits of the ASC-1 complex can also associate in a different way to fulfill other activities (Figure 3). Thus, ASC-1 or ASCC1 appeared dispensable in two regulatory processes contributing to DNA damage response and resolution of ribosome stalls [36,37,39,40].

The smallest ASCC complex has been discovered while studying a new molecular mechanism coupling a helicase and a dealkylating enzyme to perform DNA damage repair and maintain genomic integrity [38]. An unbiased proteomic approach identified ASCC1, ASCC2 and ASCC3 as the subunits associated with this DNA alkylation repair complex. The latter acts as a sensor of a new ubiquitin-dependent signaling involved in DNA repair, resulting in the recruitment of alkylation repair enzymes, ASCC3 and ALKBH3. The ubiquitin binding and the helicase activities of ASCC2 and ASCC3, respectively, were essential to the function of this alkylation damage signaling pathway [37]. So far, the proper assembly of this ASCC complex appears to rely on ASCC1, which also contributes to address the ASCC complex to alkylated nucleotides foci via a mechanism that still needs to be clarified [36].

A third complex containing ASCC3, ASCC2 and ASC-1 is associated with abnormal ribosome stalling resolution. Collided ribosomes marked with ubiquitin trigger the formation of the so-called human Ribosome-associated Quality Control (RQC)-Trigger complex (hRQT). Dedicated to quality control of translation, this complex ensures accurate gene expression and maintenance of protein homeostasis. Recognition and disassembly of stalled ribosomes are achieved, respectively, through ASCC2 ubiquitin binding activity and ASCC3 helicase activity. The role of ASC-1 is less well defined. However, lack of ASC-1 causes unstable association of the hRQT complex with ubiquitinated ribosomes, resulting in lower efficiency of RQC induction. Therefore, ASC-1 is suspected to play a critical role in the stabilization of this association through a potential RNA binding activity [39]. In a recent study, authors showed that ASCC1 was not required for RQC induction and that this novel hRQT complex can form without ASCC1. However, another article extended the composition of the hRQT complex to the four subunits of the ASC-1 complex, illustrating the difficulty to determine the functional minimal complex required for this activity [40].

The main difference between these transcriptional, post-transcriptional or translational activities appears to be their subcellular localization: RQC is performed in the cytoplasm whereas transcriptional and post-transcriptional regulation occurs in the nucleus. This clearly suggests that each individual subunit of those complexes may localize in both the cytoplasm and the nucleus. Up to date, evidence concerning their potential shuttling between both cellular compartments is poor. ASC-1 has been found by indirect immunofluorescence to be a nuclear protein that localizes into the cytoplasm under conditions of serum deprivation [41], but more recent studies in human fibroblasts did not confirm this point [23].

ASCC2 and ASCC3 appear as the functional core common to the 3 complexes described above (see Figure 3). Moreover, ASCC3 may be essential in the occurrence of the 3 different complexes, since its depletion by silencing was reported to reduce levels of expression of all the other components [40]. According to our current knowledge, the functional differences between those complexes appear to rely on the presence or absence of the last two subunits, ASCC1 and ASC-1. They contribute to the localization or the assembly of the complexes and they also play a critical role in stabilizing interactions involving a broad range of partners such as ribosomes, elements of the RNA polII machinery, transcription factors or transcriptional regulators.

Consequently, their absence or any alteration in their structure might lead to pathological outcomes. For instance, the deficit in ASC-1 protein observed in CM patients may impact the formation of some complexes causing a loss of function, with expected outcomes in essential cellular processes such as proliferation-differentiation, migration or cell survival, and RNA metabolism and translation. By contrast, shorter forms of ASC-1 could induce other types of complex dysfunction with impact on neuromuscular development and bone metabolism. Much remains to be elucidated regarding the function of the individual subunits of the ASC-1 complex as few functional domains have been identified so far.

## 6. ASC-1 Is a Novel Regulator of Cell Proliferation, Growth and Cell Cycle

While the specific role of ASCC1 in the neuromuscular system has not been experimentally studied so far, ASC-1 has emerged in the last years as a novel regulator of several cell mechanisms and pathways which are pivotal for muscle development and growth.

The formation of skeletal muscle (myogenesis) is a sequential process which includes the proliferation of myogenic mononucleated cells, followed by induction of muscle-specific gene expression, exit from the cell cycle and fusion to form multinucleated myotubes which subsequently will grow to become differentiated muscle fibers [42]. Using patient-derived cultured muscle cells, we found that ASC-1 absence impairs the growth of myotubes. This was confirmed by in vitro experiments using a *Trip4* knockdown (*Trip4*KD) murine myogenic cell line, which showed a reduced myotube diameter and a significant decrease in the expression of sarcomere proteins such as myosin heavy chain. Both ex vivo and in vitro results contributed to identify ASC-1 as a new regulator of late myogenic differentiation and myofiber growth [18]. This work established for the first time that ASC-1 is indispensable for human skeletal muscle, identified transcriptional co-regulation as a novel pathophysiological pathway and suggested defects in myotube growth as a novel myopathic mechanism.

The complex mechanisms that regulate muscle fiber growth are still incompletely understood, and identifying a novel regulatory protein is interesting progress. However, part of the cell growth is determined at pre-differentiation (proliferation) stages. Thus, using the aforementioned *Trip4*KD C2C12 cell line, we investigated the role of ASC-1 in cell proliferation. We found increased myoblast proliferation in the absence of ASC-1 due to a shortening of the G0/G1 phase of the cell cycle, leading to acceleration of the cell cycle progression and reduction in myoblast size [22]. This cell phenotype was associated with altered expression of proteins controlling the progression of the cell cycle. Levels of cyclin D1 and p21, two leading actors of the cell cycle G1 phase, were significantly increased in the absence of ASC-1, suggesting an increased formation of the cyclin D1-Cdk4/6 complex responsible for phosphorylation the Retinoblastoma protein (pRb). This master cell-cycle control protein also regulates muscle-specific gene expression through its target E2F. Hypophosphorylated pRb inhibits E2F transcription factors required for G0/G1-S transition and S phase entry. Indeed, ASC-1 depleted cells showed a strong tendency towards a decrease in the hypophosphorylated forms of pRb in favor of hyperphosphorylation. Thus, cyclin D1 and p21 overexpression in the absence of ASC-1 induced a hyperphosphorylation of pRb which possibly leads to loss of pRb-mediated inhibition of the G0/G1-S transition, to cell cycle acceleration and to reduced cell growth. Increased cell proliferation was also confirmed in primary skin fibroblasts derived from *TRIP4*-mutant patients, which is consistent with the presence of skin abnormalities in ASC1-RM [22] and indicates that ASC-1 is involved in cell cycle regulation in both fibroblasts and myogenic cells. As proliferation is a basal physiological process, this cell phenotype could have implications also for other extra-muscular tissues including adipose tissue, whose distribution was altered in some ASC1-RM patients [18,22].

Correlations between cell cycle and cell size and/or growth have long been studied in budding yeast and more recently in mammalian cells [41,43]. Increasing evidence suggests that alterations in regulatory mechanisms of cell size control may impact protein synthesis threshold in particular cell types [43], and might contribute to explain the decreased expression of some key myogenic proteins observed in the *Trip4*KD C2C12 model. Additionally, pRb also regulates late muscle development, promoting myofibrillogenesis and muscle cell growth by activating transcription of myogenic and metabolic genes. Consistently, we also found an abnormal expression of cyclins in post-mitotic frozen muscle biopsies from patients with *TRIP4*-mutations leading to ASC-1 absence [22]. Thus, these mechanisms are not only relevant for the late myogenic differentiation and myofiber growth defects reported in ASC-1 devoid myotubes, but might contribute as well to explaining the various myofibrillar defects observed in patients’ biopsies.

**Figure 4:**
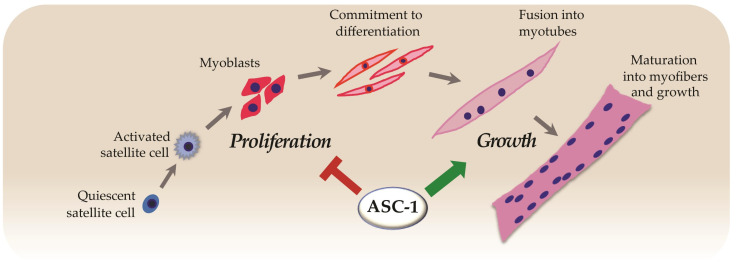
ASC-1 modulates cell cycle progression in proliferative myogenic cells by regulating cell cycle protein expression and phosphorylation [22] and promotes muscle cell growth [18].

Taken together, the current data suggest that ASC-1 regulates at least two steps of myogenesis, as a negative cell-cycle regulator of proliferation and as a key player in promoting the growth of both proliferating myoblasts and differentiated myotubes (Figure 4).

## 7. Discussion

In the last years, identification of inherited defects in the ASC-1 complex and their association with monogenic congenital neuromuscular disease has represented a significant progress in the molecular characterization of these rare disorders, and revealed defects in co-transcriptional regulation, cell cycle and cell growth as novel pathomechanisms. However, it has also raised interesting questions which remain so far unanswered and represent interesting avenues of future investigation. Transcriptional cointegrators act as coactivators or corepressors through the integration of transcription factors or nuclear receptors in multi-protein complexes, and can thereby adapt cell metabolism to environmental changes or modulate gene expression in a tissue-specific way [44]. Thus, unraveling their physiological role can help to understand a variety of physiological pathways, and to clarify why inherited defects of a ubiquitous protein are often associated with a tissue-specific phenotype.

An important step would be establishing definitely which tissues and systems are affected in individuals with *TRIP4* or *ASCC1* mutations, namely clarifying the puzzling question of motoneuron versus primary muscle involvement. The answer to this might not be a simple one, since antenatal development of motoneurons and muscle are bidirectionally interdependent; full maturation of muscles requires adequate contact with motoneuron endplates, and conversely retrograde signaling from muscle towards motoneurons contributes to motor neuron survival and synapse maturation at postnatal stages [45]. Thus, primary defects in either motoneurons or muscle fibers can lead to a structurally and/or functionally abnormal neuromuscular junction (NMJ) and to overlapping phenotypes, particularly difficult to differentiate in newborn patients.

So far, 6 out of the 9 reported families with *TRIP4* mutations presented clinically, electrophysiologically and histopathologically with a primary skeletal muscle phenotype, associated in some cases with cardiac muscle disease (cardiomyopathy), and had severe depletion or absence of ASC-1 protein. Thus, the most common phenotype associated hitherto with *TRIP4* defects is a congenital myopathy termed ASC-1 related myopathy. Strikingly, three families sharing two *TRIP4* mutations which led to upregulation of a shorter ASC-1 isoform presented with a more severe antenatal phenotype. Muscle biopsies from these patients disclosed immature muscle fibers of reduced and variable size, which is compatible with a primary muscle disease and with the reported role of ASC-1 in promoting muscle fiber growth. The cardiomyopathy in two of these three families also supports a potential primary striated (skeletal and cardiac) muscle involvement. However, in some patients from these severe families, grouping of type 1 fibers (classically a neuropathic sign), neurography studies suggesting axonal neuropathy and/or, for one patient, apoptotic spinal cord α-motoneurons were reported. These findings prompted the diagnosis of a primary motor neuron disease. Interestingly, knock-down of *trip4* in zebrafish led both to perturbed outgrowth of α-motoneuron axons and to abnormal muscle structures (myotomes with severely reduced myofibril formation), together with defective NMJs [23]. These findings could be secondary to a neuronopathy with downstream effects on NMJ and muscle, but might also be compatible with a retrograde involvement of NMJ and motoneurons secondary to a primary muscle defect. Actually, both mechanisms could coexist, although it cannot be excluded at this point that different types of *TRIP4* mutations are associated with preferential involvement of different tissues. Thorough studies of additional cases with other *TRIP4* mutations, including deep-phenotyping (electromyography, muscle biopsy, neuroimaging), ASC-1 protein studies and careful phenotype-genotype correlations, will hopefully contribute to clarify this point.

The situation is not very different regarding *ASCC1.* Patients from the seven reported families with *ASCC1* mutations share a common clinical phenotype, but for some of these families, muscle biopsy or electromyographies have not been performed. Böhm and colleagues studied exhaustively three families with *ASCC1* mutations, reporting myopathic histopathological and electromyographic features without signs of motor neuron involvement. Thus, they considered ASCC1-related disease as a severe congenital myopathy [24]. In contrast, the family described by Knierim et al. had, aside from major reduction in fiber size, fiber type grouping and neurogenic EMG. In a more recent case, muscle biopsy was described as atrophic with no further description [25]. However, unlike in *TRIP4*-mutant patients, brain MRI abnormalities were observed in two families with ASCC1 defects and myocardial involvement has never been reported, which might support a stronger association of ASCC1 defects with neuronal involvement. Furthermore, transcriptomic studies in skin fibroblasts from one *ASCC1*-mutant patient showed downregulation of genes associated with neurogenesis, neuronal migration, and pathfinding, as well as with bone development [23].

In fact, a growing body of evidence suggests that the ASC-1 complex could be involved in neurodegenerative pathomechanisms. The ASC-1 complex associates with the RNAP II machinery and ALS or SMA-causative proteins [25]. Besides, known targets of the ASC-1 complex including NF-kB and AP-1 transcription factors have been recently linked to ALS [46,47,48]. Furthermore, the ASC-1 complex contributes to counteracting defective DNA damage repair (DDR), one of the mechanisms underlying neurodegenerative disorders [49,50]. Neurons are highly metabolically active, produce great amounts of ROS and usually lack the homologous recombination pathway of DDR, all of which can potentially result in neurodegeneration due to accumulation of DNA damage [51,52]. The latter includes DNA alkylation [53] requiring a dealkylation repair complex whose formation, as we previously mentioned, is regulated by ASCC1 [36].

Congenital bone fractures, present in only two families with *TRIP4* mutations [23], are part of the clinical phenotype in all the *ASCC1*-mutated patients [24,33,34,35]. Although prenatal fractures have been non-specifically reported in other forms of congenital muscle disease [54,55,56], their frequency in *ASCC1*-mutant cases suggests that, in this context, they are not an unspecific consequence of fetal immobility but directly linked to a role of ASCC1 in bone. Interestingly, ASCC1 absence has been shown to inhibit AP-1 transactivation by the ASC-1 complex [19], and several transcription factors from the AP-1 family were recently shown to play a role in bone development [57,58,59,60]. Furthermore, down-regulation of osteoprogenitor proliferation is a critical step for osteoblast differentiation, and several cell cycle regulators such as p27 [61] or Cdk6 [62] contribute to growth and differentiation of osteoblasts. This suggests that ASCC1 might regulate proliferation and differentiation of bone progenitors, its defects resulting in abnormal bone structure.

Indeed, our data suggest a potential function of the ASC-1 complex in modulation of the balance between cell proliferation and cell differentiation in different tissues [18,22]. Deregulation of cell proliferation is a hallmark of cancer. Germline mutations in the *ASCC1* gene have been associated with Barrett esophagus and esophageal adenocarcinoma [63,64]. It has been shown that *TRIP4* is highly expressed in human melanoma [65], cervical cancer [66] and colorectal cancer [67] cell lines and tumor tissues, and that *TRIP4* knockdown reduces proliferation, migration and invasion of tumor cells. The latter seems in contradiction with our data showing negative regulation of cell proliferation by ASC-1 in non-cancerous cells. However, it is conceivable that the ASC-1 complex, as a transcriptional co-integrator, does not only have a unidirectional direct role but, depending on the cell environment and on its association with different proteins, can differentially modulate diverse pathways in different cell types. Thus, ASC-1 can modulate estrogen receptor-α [68], essential for proliferation, in breast cancer cells, activate MAPK and PI3K/AKT signaling in cervical malignant cells [66] or co-regulate the expression of proinflammatory cytokines COX-2/iNOS with p300 in melanoma cells [65]. Further functional studies are needed to characterize fully its functional role in human cancer.

In conclusion, the transcriptional coregulator ASC-1 complex is emerging as a key player in several major physiological and pathophysiological processes. We recommend that *TRIP4*, *ASCC1* and potentially the genes encoding the other protein components of the ASC-1 complex should be systematically included in diagnostic gene panels of congenital neuromuscular, myocardial and/or bone diseases. Further studies including deep phenotyping and careful phenotype-genotype correlations in additional patients with new inherited defects of ASC-1 complex proteins are mandatory in order to establish a diagnosis of congenital myopathy or spinal muscular atrophy. Further functional studies will hopefully contribute to a clarification of the role(s) of the ASC-1 complex and provide additional illustration of the interest of mechanistic studies of rare or ultra-rare monogenic disorders as a useful model paradigm to understand complex pathophysiological pathways underlying many different prevalent human conditions, including malignant or neurodegenerative diseases.

## Figures and Tables

**Table 1:** Summary of the 10 *TRIP4* mutations identified so far. Some patients were compound heterozygous (comp het) for a missense and a truncating mutation (in exons 4 + 11) or for two different truncating mutations (in exons 1 + 9 or 6 + 7).

Publications	*TRIP4* Exon	cDNA Change	Protein Change	Variation Type	Predicted Effect	Primary Involvement Reported
Davignon et al., 2016	7	c.950G>A (homozygous)	p.Trp297Ter	substitution	nonsense	muscle
Knierim et al., 2016	6 | 7	c.760C>T (hom or comp het)	p.Arg254Ter	deletion	nonsense	motor neuron + bone
		c.832C>T (hom or comp het)	p.Arg278Ter	deletion	nonsense	motor neuron + bone
Villar-Quiles et al., 2020	2	c.141_142delAT (homozygous)	p.Tyr48CysfsTer3	deletion	frameshift	muscle
	4 | 11	c.534C>G (comp het)	p.His178Gln	substitution	missense	muscle
		c.1544_1547delACTG (comp het)	p.Asp515AlafsTer34	deletion	frameshift	muscle
	8	c.1065delC (homozygous)	p.Ile356LeufsTer6	deletion	frameshift	muscle
	1 | 9	c.55_56insCT (comp het)	p.Gln19ProfsTer47	insertion	frameshift	muscle
		c.1197delA (comp het)	p.Ser399SerfsTer12	deletion	frameshift	muscle
	8 + 9	homozygous deletion exons 8 and 9		deletion	in-frame deletion	muscle

**Table 2:** Summary of the 7 *ASCC1* mutations identified so far. Some patients were compound heterozygous (comp het) for a nonsense mutation associated with a nucleotide duplication (in exons 3a and 5, respectively) or with microdeletions (in exons 9a and 6-9a or in exons 5 and 9). The localization of the mutations reported in the table corresponds to those mentioned in each article. Authors generally used the exon nomenclature from the first description by Knierim et al. [23] with the exception of Lu et al. [35]; n.c: non-characterized.

Publications	*ASCC1* Exon	cDNA Change	Protein Change	Variation Type	Predict Effect	Primary Involvement Reported
Knierim et al., 2016	3a	c.157dupG (homozygous)	p.Glu53GlyfsTer19	single base pair duplication	frameshift	motor neuron/CNS + bone
Oliveira et al., 2017	3a	c.157dupG (homozygous)	p.Glu53GlyfsTer19	single base pair duplication	frameshift	n.c. muscle? + bone
Böhm et al., 2019	3a | 5	c.157dupG (hom or comp het)	p.Glu53GlyfsTer19	single base pair duplication	frameshift	muscle + bone
		c.466C>T (comp het)	p.Arg156Ter	substitution	nonsense	muscle + bone
	6	c.667C>T (homozygous)	p.Glu223Ter	substitution	nonsense	muscle +bone
Giuffrida et al., 2019	9a | 6–9a	c.1027C>T (comp het)	p.Arg343Ter	substitution	nonsense	bone + ?
		hemizygous deletion exons 6 to 9a		microdeletion	in-frame deletion	bone + ?
Lu et al., 2020	5 | 9	hemizygous deletion in exon 5		microdeletion	frameshift	CNS + bone
		c.932C>G (comp het)	p.Ser311Ter	substitution	nonsense	CNS + bone

## Abbreviations

ALKBH3	Alpha-ketoglutarate-dependent Dioxygenase alkB Homolog 3
ALS	Amyotrophic Lateral Sclerosis
AP-1	Activator Protein 1
ASC1-RM	ASC-1 Related Myopathy
ASC-1	Activating Signal Cointegrator-1
ASCC1	Activating Signal Cointegrator-1 complex subunit 1
ASCC2	Activating Signal Cointegrator-1 complex subunit 2
ASCC3	Activating Signal Cointegrator-1 complex subunit 3
ASCC	Activation Signal Cointegrator Complex
ASCH domain	ASC-1 homology domain
CDK4/6	Cyclin-dependent kinase 4 and 6
CM	Congenital Myopathy
CNS	Central Nervous System
COX-2	Cyclooxygenase 2
CUE	Coupling of ubiquitin to ER degradation
DDR	DNA Damage Response
EM	Electron Microscopy
EMG	Electromyogram
ER	Endoplasmic Reticulum
G0	quiescent phase of the cell cycle
G1	gap1 phase of the cell cycle
KH domain	K-homology domain
MAPK	Mitogen Activated Protein Kinase
MHC	Myosin Heavy Chain
MRI	Magnetic Resonance Imaging
mRNA	messenger RNA
NADH-TR	NADH-tetrazolium reductase
NF-kB	nuclear factor-kappa B
NOS	Nitric Oxide Synthase
PI3K	Phosphoinositide 3 Kinase
pRb	Retinoblastoma protein
RNAP II	RNA polymerase II
ROS	Reactive Oxygen Species
RQC	Ribosome-associated Quality Control
hRQT	human RQC-trigger complex
SMA	Spinal Muscular Atrophy
snRNP	small nuclear ribonucleoprotein
SRF	Serum Response Factor
TRIP4	Thyroid Hormone Receptor Interactor 4
